# The antioxidant, anti-cholangiocarcinoma, and anti-*Opisthorchis viverrini* activities of ethanolic extract from *Antidesma thwaitesianum* fruit

**DOI:** 10.5455/javar.2024.k813

**Published:** 2024-09-29

**Authors:** Ratchadawan Aukkanimart, Pranee Sriraj, Areeya Changthong, Tichanon Promsrisuk

**Affiliations:** 1 Department of Thai Traditional Medicine, Faculty of Natural Resources, Rajamangala University of Technology Isan, Sakon Nakhon, Thailand; 2 Cholangiocarcinoma Research Institue, Khon Kaen University, Khon Kaen, Thailand; 3 Division of Physiology, School of Medical Sciences, University of Phayao, Phayao, Thailand

**Keywords:** Anti-cholangiocarcinoma, cyanidin-3-O-glucoside, *in vivo*, opisthorchis *viverrini*, reducing inflammation

## Abstract

**Objectives:**

The current study was performed to determine the antioxidant, anti-inflammatory, and anti-cholangiocarcinoma (CCA) properties of *Antidesma thwaitesianum,* also known as “MAO,” whole plant extract on Opisthorchiasis in animal models and CCA cell lines.

**Materials and Methods:**

Ethanol was used to extract compounds from the whole ripe fruit. The phytochemical investigation of MAO extract was done to evaluate antioxidant activity, and high-performance liquid chromatography was used to identify the active compounds. The efficacy of MAO extract against OV was evaluated *in vivo*. The anti- CCA activity was evaluated using superoxide dismutase (SRB), cell cycle arrest, apoptosis, and western blot analyses.

**Results:**

MAO extract possessed flavonoid and phenolic contents, antioxidant activity, and an expressed cyanidin-3-O-glycosides content of 0.08 μg/mg extract. MAO extract demonstrated hepatoprotective effects through raised alanine transaminase and alkaline phosphatase levels, as well as an influence on oxidative stress via decreased MDA and increased glutathione, superoxide dismutase, and catalase levels. MAO extract significantly inhibited the migration of CCA cells in a concentration- and time-dependent manner, as well as triggered cell cycle arrest on G1 and activated apoptosis pathways via upregulation of C3, downregulation of cyclin-dependent kinase inhibitor p21, cyclin D, and cyclin-dependent kinases 2 expression. MAO extract inhibited inflammation, which in turn decreased fibrosis in hamsters. It also increased hepatoprotective activity.

**Conclusion:**

Our findings demonstrate the potential benefits of MAO extract in both *in vitro* and animal studies of hamster opisthorchiasis. However, more research should be done to ascertain the mechanisms of activity of MAO extracts and elute bioactive components in order to confirm their safety and examine their clinical applications.

## Introduction

The north-eastern region of Thailand, especially Sakon Nakhon, Nakhon Panom, and Khon Kaen provinces, has the highest worldwide incidence of CCA (0.85%), with the northern, central, and southern regions of Thailand following [1]. CCA progression is caused by parasitic diseases such as infection with liver flukes, *Opisthorchis viverrini* (OV) [2]. Currently, the side effects of anti-cancer agents have also increased. Most pharmacological drugs with anticancer properties affect the step of cell division. It is noteworthy that specific normal cells, such as those found in the bone marrow, oral mucosal cells, and hair follicles, multiply aggressively alongside cancer cells [3]. The symptoms in patients receiving chemotherapy are oral problems, insomnia, psychological disorders, fatigue, and severe loss of appetite [4].

To date, researchers have been studying natural products on their anticancer activity and ability to reduce the side effects of cancer therapies [5]. *Antidesma thwaitesianum* Muell. Arg (*Phyllanthaceae*), MAO, is a native fruit tree of northeastern Thailand. *Antidesma thwaitesianum* fruits are processed to make juice, wine, jam, marcs, and so on. These products contain significant anthocyanins like cyanidin-3-glucoside, delphinidin 3-O-glucoside, ellagic acid, and so on. Several health advantages have been demonstrated, including liver fat metabolism, anti-inflammatory and antioxidant properties, as well as the suppression of carbohydrate-digesting enzymes [6–8]. There are, however, no reports on the application of MAO extracts to cancer cells and liver fluke infections. The purpose of this study is to investigate the antioxidant, anti-inflammatory (*in vivo* opisthorchiasis), and anti-proliferation (*in vitro*) properties of MAO extracts.

## Materials and Methods

### Ethical approval

All protocols of animal handling were authorized by Khon Kaen University’s animal ethics committee (ACUC-KKU 14/2560).

### Chemicals and reagents

Analytical-grade materials were used for all compounds and reagents in this study. The following items were purchased from RCI Labscan Asia (Thailand): ethanol, formic acid, and hydrochloric acid. Folin-Ciocalteu reagent was obtained from Loba Chemie in India. Gibco (USA) provided the bovine serum (FBS), RPMI-1640, penicillin-streptomycin, amphotericin B, and trypsin- ethylenediamine tetraacetic acid. Sulforhodamine B, trichloroacetic acid, and potassium peroxodisulfate were obtained from Sigma-Aldrich in Switzerland. Wako (Japan) provided the 2, 2-diphenyl-1-picrylhydrazyl (DPPH), ABTS (2, 2-azino-bis (3-ethylbenzothiazoline-6-sulphonic acid), and Annexin-V-FLUOS staining kit from Roche (Germany). RNase and propidium iodide (PI) were bought from BD Biosciences (USA).

### Plant components and extraction

**Figure 1.**
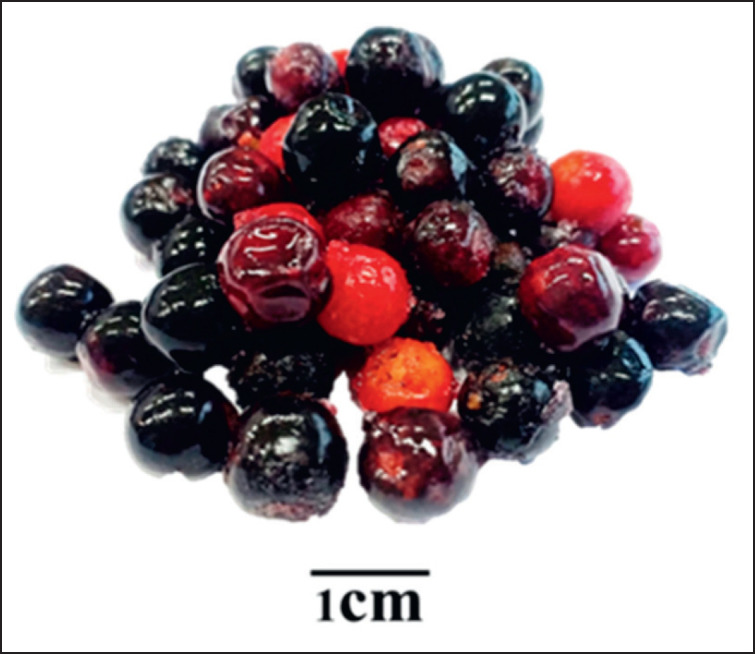
Antidesma thwaitesianum fruit.

MAO, Faprathan cultivar ripe fruit (*A. thwaitesianum*), was obtained from Phuphan District in Sakon Nakhon province, Thailand. Assist. Prof. Pichet Wetwithan identified the MAO species. The plant voucher was taken to the herbarium of the Department of Thai Traditional Medicine; their accession number is Ath202001 (Fig. 1). The extraction was done according to a previous study. Briefly stated, 100 gm of ripe fruit were soaked in a solution of 70% ethanol and 0.1% HCl (1:10 w/v) for 24 h before being evaporated using a rotavap (Buchi, Switzerland). The crude extracts were then kept at −20^o^C for subsequent examination [9].

### High-performance liquid chromatography (HPLC) analysis

A Shimadzu HPLC system was employed to evaluate the MAO extracts. A C18 column (3.9 × 150 mm) (Waters, USA) was used for reverse phase separation at 40°C. The isocratic mobile phase contained methanol and distilled water (80:20) at a flow rate of 1.0 ml/min. Each extract was evaluated in triplicate at a concentration of 1 mg. Cyanidin-3-O-glucoside (C3G) (C_21_H_21_C_l_O_11_, 97% purity, Sigma-Aldrich) was diluted with methanol to provide concentrations of 0.78, 1.56, 3.125, 6.25, 12.5, 25, and 50 μg/ml. The following HPLC operating conditions were used: injection volume of 10 μl, column flow rate of 1.0 ml/min, chromatographic system run duration of 15.0 min, and PDA recording of spectra at 254 nm.

### Determination of total phenolic content (TPC) and total flavonoid content (TFC)

The folin-Ciocalteu technique and gallic acid standard equivalent were used to quantify TPC. The MAO extract, 1 mg/ml, was combined with 20 μl Folin-Ciocalteu reagent, 160 μl distilled water, and 100 μl 7.5% sodium carbonate. The solution was then measured at 765 nm, and a calibration curve for gallic acid was constructed. TPC concentrations were calculated in milligrams of gallic acid equivalents (GAEs) per gram of extract [10]. The colorimetric technique for aluminum chloride was done following the method described by [11]. Briefly, 50 μl of 1 mg/ml MAO extract and 50 μl 2% aluminum trichloride (AlCl_3_) solution were incubated at room temperature. The extracts’ absorbance was quantified at 435 nm, and the TFC was expressed in mg quercetin equivalents (QEs)/g extract.

### Antioxidant activity

The procedure in [12] was followed for the DPPH technique. In summary, 100 μl of the MAO extract (0–1,000 μg/ml) was combined with 100 μl of 0.2 mM DPPH solution, and the mixture was left at room temperature in the dark for 30 min. Next, using a microplate reader (Tecan, Switzerland), the absorbance was determined at 517 nm. A modified method from [10] was used for the ABTS radical scavenging activity. The reaction mixture was made up of a 7. mM ABTS stock solution and 2.45 mM potassium persulfate that was left to stand for 12 to 16 hours. After ethanol was added, the resulting ABTS solution was diluted to an absorbance of 0.70 ± 0.02. For each test, fresh ABTS+ solution, 150 μl of ABTS+ solution, and 50 μl of MAO extract were used, and the samples were kept at room temperature for 30 min. Spectrophotometric measurement of the absorbance was performed at 734 nm using a microplate reader (Tecan, Switzerland).

### In vivo hamster opisthorchiasis and treatment

In summary, *O. viverrini* metacercariae were collected from cyprinid fish in Laos PDR. Fish was minced in a 0.25% acid pepsin solution, incubated at 37°C for 1 h, and then filtered through 1000, 300, 250, and 106 m sieves. The sample was sedimented with 0.85% saline repeatedly until a clear precipitate was obtained and examined for the abundance of *O. viverrini* metacercariae. A parasitologist identified and collected infectious metacercariae using a dissecting microscope. Fifty OV metacercariae were given to each hamster via stomach intubation. The Animal Care Unit, Faculty of Medicine, Khon Kaen University provided male Syrian hamsters (7 weeks old). A commercial diet (C.P. Ltd., Thailand) was fed ad libitum to the hamsters, who were housed in cages, with 5 hamsters per cage.

### Experimental design

Hamsters (*n = *20) were categorized into four groups (*n = *5):

- Group1 (I) normal control treated with distilled water (Normal) 1 ml/kg body weight;
- Group2 (II) normal control treated with MAO 100 mg/kg (MAO);
- Group 3(III) *O.* *viverrini* infected (OV); and
- Group 4 (IV) *O.* *viverrini* infected and treated with MAO 100 mg/kg (OVM).

The treatment was orally administered daily according to the experimental design for 1 month.

### Sample collection for biochemical analysis

Hamsters were CO_2_-euthanized, and entire blood was obtained directly from the heart. The sera were tested for liver and renal function, and the livers were removed and used for biochemical and histological assessments. Blood chemistry tests were conducted on sera to determine levels of alanine aminotransferase (ALT), alkaline phosphatase (ALP), blood urea nitrogen (BUN), and creatinine using an established kit (Thermo Fisher Scientific, USA). Spectrophotometry (Automate RA100; Thermo Trace Ltd., Australia) was conducted at the Faculty of Associated Medical Sciences, Khon Kaen University. Malondialdehyde (MDA), glutathione (GSH), superoxide dismutase (SOD), and catalase (CAT) contents in plasma were used to measure the level of oxidative stress, which were determined using spectrophotometry according to a previously published technique [13,14]. In summary, for the MDA assay, 150 μl of plasma was treated with 10% trichloroacetic Acid (TCA), EDTA, SDS, butylated hydroxytoluene, and tertiary butyl alcohol. The mixture was heated in a water bath. A spectrophotometer was used to determine the supernatant’s absorbance at 532 nm. A standard curve was created with acceptable doses of 1,1,3,3-tetraethoxypropane (0.3–10 M). A previously established approach utilizing M2VP as a GSH scavenger was used to assess total glutathione in the samples. We immediately reacted the samples with either M2VP or distilled water, then treated them with 5% MPA to precipitate the protein. Following centrifugation, the supernatant was used in the enzymatic coupling test for GSH using a spectrophotometer (Biochrom Ltd., Cambridge, UK). The GSH/GSSG redox ratio was determined. A commercial kit was used to estimate SOD activity. This is based on the O_2_ formed by xanthine and xanthine oxidase combined with phenyl tetrazolium chloride to generate a red formazan dye, which is used to detect certain substances. The level of response inhibition in sera was used to gauge RBC-SOD activity. This is predicated on the capacity of SOD to inhibit superoxide, which is generated during the oxidation-reduction process of riboflavin and oxygen, from reducing nitroblue tetrazolium. CAT activity was assessed by observing the decline of H_2_O_2_. Pipetting was used to completely mix 100 μl of serum with H_2_O_2_ in phosphate-buffered saline. At 1-minute intervals, the absorbance was directly observed at 240 nm for 3 min. Each sample’s CAT activity was assessed, and the average rate was expressed in mAU/min/mg protein.

### Histopathology staining analysis

Briefly stated, liver tissue was removed and promptly preserved in 10% buffer formalin for 24 h. Paraffin wax was used to embed liver tissue samples so they could be cut into 4 μm sections with a microtome. After being soaked in a succession of xylene and alcohol, tissue sections were stained using Harris’ hematoxylin and eosin and Sirius red stain technique [15].

### Cell line and culture

Human cholangiocarcinoma (CCA) cells (KKU-214; moderately differentiated adenocarcinoma) were provided by the Cholangiocarcinoma Research Institute, Khon Kaen University. Cell lines were grown in RPMI medium supplemented with 10% inactivated FBS, penicillin, and amphotericin B in a 5% CO_2_ incubator at 37^o^C.

### sulforhodamine B (SRB) assay

Examining growth inhibition on KKU-214 cell lines was done using the SRB assay. An amount of 3 × 10^4^ cells per well was used for the planting of cells. After incubating for 24 h, the cells were exposed to MAO extracts for 24–72 h at 0–1,000 μg/ml. After 10% TCA was used to fix the cells, 0.4% SRB was used to dye them. Utilizing a microplate reader (TECAN, Austria), spectrophotometric measurement of the absorbance was carried out at 510 nm. The percentage of cell viability was computed. The IC_50_ value was obtained using Calcusyn 3.0.

### Flow cytometry

KKU-214 cells were treated with MAO extracts [doses 30% maximal inhibitory concentration (IC_30_), 50% maximal inhibitory concentration (IC_50_), and 80% maximal inhibitory concentration (IC_80_] for 24 h (5 × 10^5^ cells/well). To analyze the cell cycle, KKU-214 cells were collected, cleaned, and fixed in 70% ethanol overnight before being rinsed with PBS, treated with 0.1 mg/ml RNase, and stained with 40 μg/ml propidium iodide (BD Biosciences, USA). The Annexin-V-FLUOS Staining Kit was used to identify apoptotic cells. In summary, after being treated with MAO extract, KKU-214 cells were stained for 15 min in the dark using propidium iodide and an Annexin V kit. Flow cytometry (FACSCanto II, BD Biosciences, UK) was used to analyze the samples.

### Western blot analysis

KKU-214 cells were lysed in a lysis buffer. A total of 50 μg of proteins were separated using 12% SDS-PAGE and transferred to a nitrocellulose membrane (Millipore, Billerica, MA, USA). The nitrocellulose membranes were blocked with 5% skimmed milk for 1 h at 37^o^C. Subsequently, they were incubated overnight at 4^o^C with primary antibodies [cyclin-dependent kinase inhibitor p21 (p21), cyclin D, cyclin-dependent kinases 2 (CDK2), caspase3, and actin] (Abcam, UK), followed by 2 h with a secondary antibody. The membranes were washed, and the proteins were detected with a Pierce Biotechnology enhanced chemiluminescence kit, quantified with ImageQuant LAS 4,000 (GE Healthcare, USA), and measured with Scion Image (Scion Corp., USA), with relative intensity estimated and standardized to actin [16].

### Statistical analysis

All data were presented as means with standard deviations (SD). The difference in means was examined using one-way ANOVA, Duncan’s multiple range test, and a non-parametric test for histopathological grading in SPSS 20.0. A *p*-value < 0.05 was considered statistically significant.

## Results

### HPLC analysis

**Figure 2.**
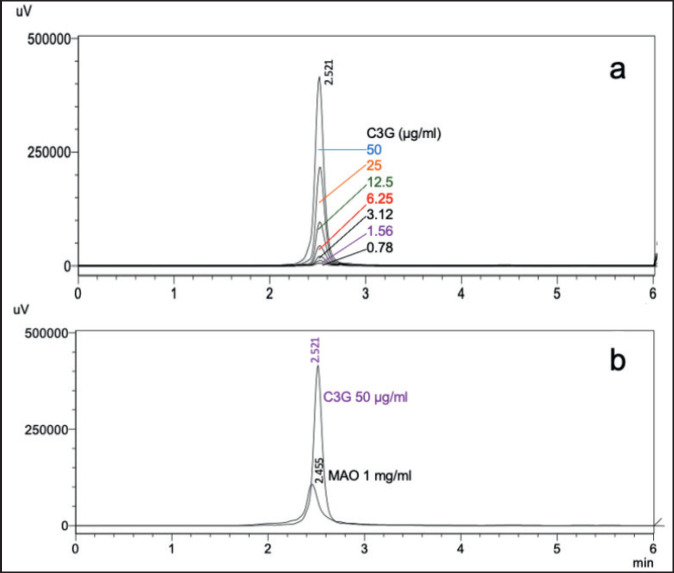
High-performance liquid chromatography analysis chromatogram. (a) C3G 0.78–50 μg/ml (b) C3G and MAO.

The compound profile of MAO extracts was determined by HPLC, as shown in Figure 2. The active chemical compounds were identified by comparing their retention times to those of the C3G standard. The R^2^ values for C3G were 0.9997, which showed a retention time (tR) of 2.521 min. The C3G content in the MAO extract was 0.08 μg/mg at tR 2.455 min.

### The phytochemical contents and anti-oxidation activity

The TPC of MAO extract was found to be 178.00 ± 11.31 mg GAE/g extract, and the amount of flavonoid was determined to be 26.73 ± 3.11 mg QE/gm extract. Antioxidant properties of MAO extract as determined using DPPH and ABTS^+^ were found to be IC_50 _37.21 ± 3.51 and 28.06 ± 2.98 μg/ml, respectively.

### OV infection, liver pathology, and hepatoprotective activity

**Figure 3.**
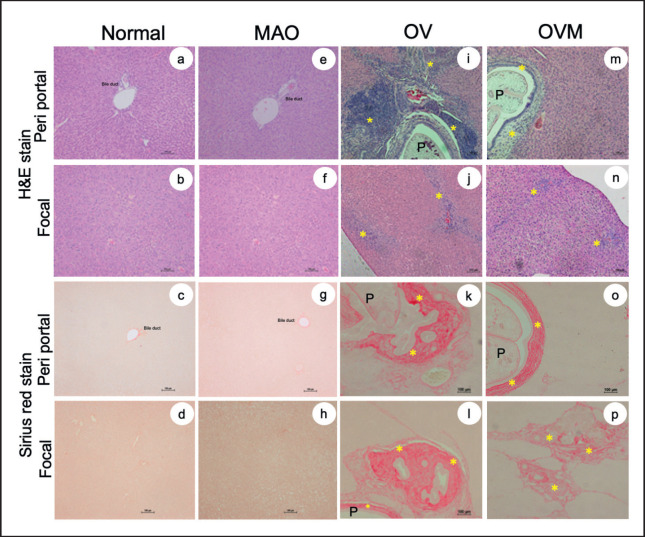
Histopathological alterations in liver tissue of normal group (a-d), MAO-treated (e-h), O.* viverrini* infected in hamsters (OV) (i-l), and OV treated with MAO (OVM) (m-p) using hematoxylin & eosin stain and Sirius red stain (P = parasite, * = inflammation and fibrosis regions).

**Figure 4.**
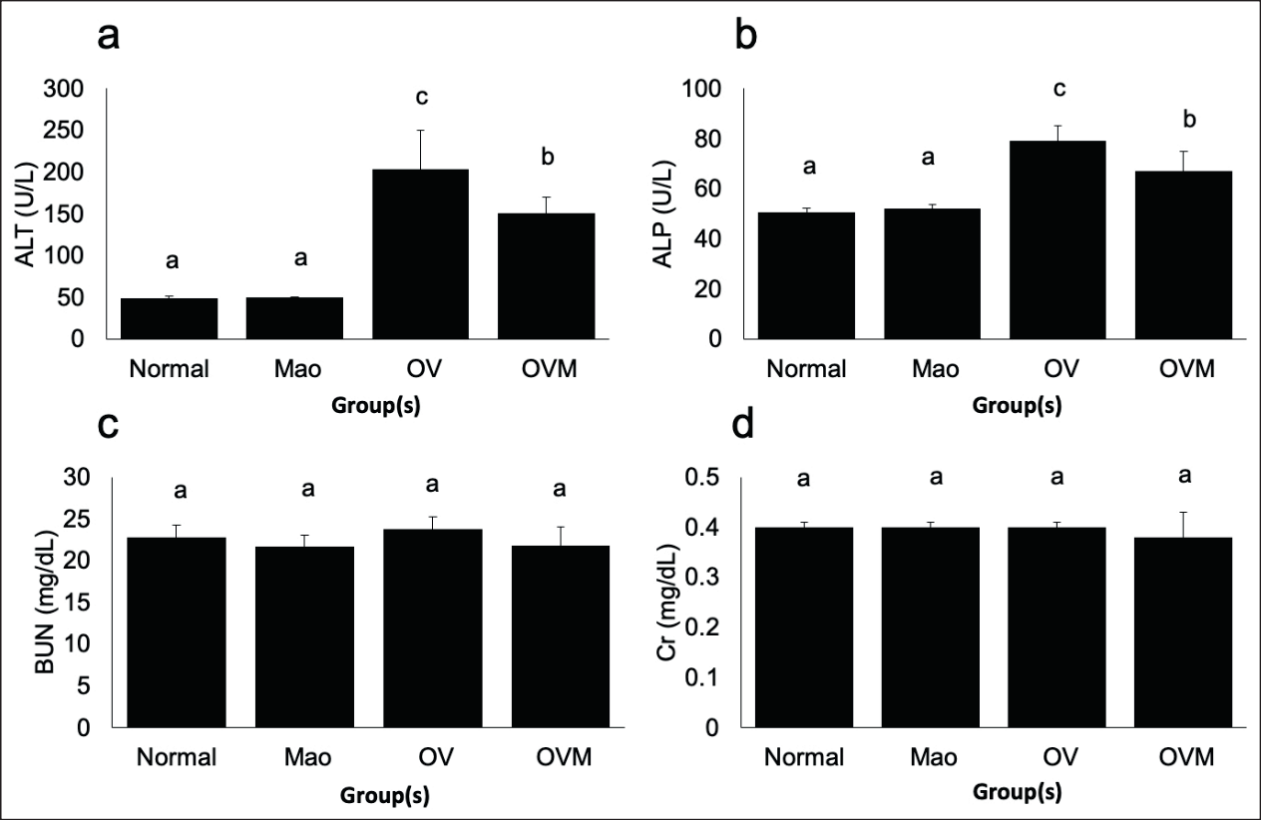
Biochemical analysis of blood samples from experimental groups; in comparison to each experimental group ^a-d^*p* < 0.05.

After 1 month of *O. viverrini* infection, inflammatory regions were discovered to be restricted to the cytoplasm of hepatocytes in the OV-infected group. Nevertheless, periportal and localized inflammation in the liver was reduced following the administration of MAO to five hamsters (Fig. 3). The treatment group *Opisthorchis viverrini* plus MAO (OVM) had lower fibrous hepatic tissue than the OV group, which corresponded to inflammatory regions (Table 1). Serum analysis was used to determine hepatoprotective efficacy. Figure 4 shows that all parameters (ALT, ALP, BUN, and Cr) of the MAO-uninfected group were comparable to the normal control group in terms of blood biochemical analysis. The ALT and ALP levels were higher in the OV-infected groups than in the OV-infected and treated with MAO extracts; however, there was no discernible difference in the two groups BUN and Cr levels.

**Figure 5.**
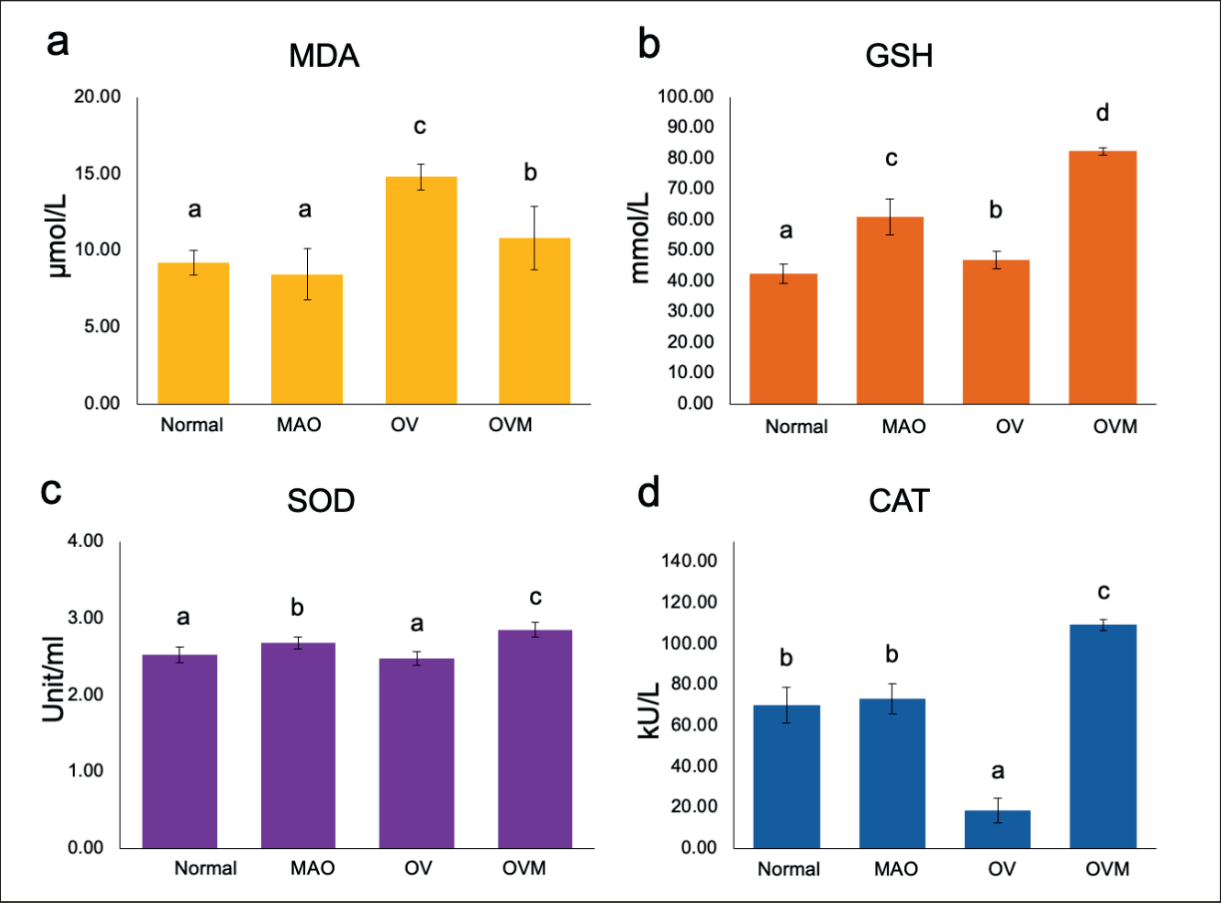
(a) MDA, (b) , (c) SOD, and (d)CAT activity in serum of hamsters in different groups: normal control, MAO, OV and OVM; in comparison to each experimental group ^a-d^*p* < 0.05.

The MDA level in the normal group was 9.22 ± 0.8 μM/l, which was similar to that of the MAO (8.45 ± 1.69 μM/l), while the OV group had 14.80 ± 0.83 μM/l, which was higher than that of the OVM group (10.83 ± 2.06 μM/l). The GSH activity in the MAO group was 61.01 ± 5.91 mM/l, which was higher than that of the normal group (42.45 ± 3.10 mM/l), while that of the OVM group was 82.34 ± 1.22 mM/l, which was higher than that of the OV groups (46.87 ± 2.83 mM/l). SOD activity in the normal group was 2.53 ± 0.10 units/ml, which was comparable to that of MAO (2.68 ± 0.07 units/ml), while that of the OVM group (2.85 ± 0.09 units/ml) was higher than that of the OV groups (2.47 ± 0.09 units/ml). The CAT level of the normal group was 70.00 ± 8.89 kU/l, which was close to that of the MAO (73.17 ± 7.38 kU/l), while the OVM group had 109.33 ± 2.75 kU/l, which was higher than that of the OV groups (18.33 ± 6.03 kU/l), as seen in Figure 5.

**Table 1.** Histopathological features of liver grading criteria at 1 month post infection.

Histopathological observation	Grade	Grading areas (*n* = 5)	
		OV	OVM
Inflammation area			
Periportal inflammation	0 1 2 3 4	5 (100%)	1 (20%)* 4 (80%)*
Focal inflammation	0 1 2 3 4	2 (40%) 3 (60%)	5 (100%)*
Fibrosis area			
Periportal fibrosis	0 1 2 3 4	1 (20%) 4 (80%)	5 (100%)*
Focal fibrosis	0 1 2 3 4	1 (20%) 4 (80%)	1 (20%)* 4 (80%)*

*p < 0.05 compared to OV group.

### MAO inhibition of CCA cell proliferation

The SRB assay was used to evaluate cell viability after exposure to MAO extract. The result revealed that MAO suppressed the growth of KKU-214 cells in a dose- and time-dependent manner. The IC_50_ values of MAO inhibition on KKU-214 cells were >100, 69.61 ± 19.45, and 140 ± 16.95 μg/ml at 24, 48, and 72 h, respectively.

### Cell cycle arrest by MAO

**Figure 6.**
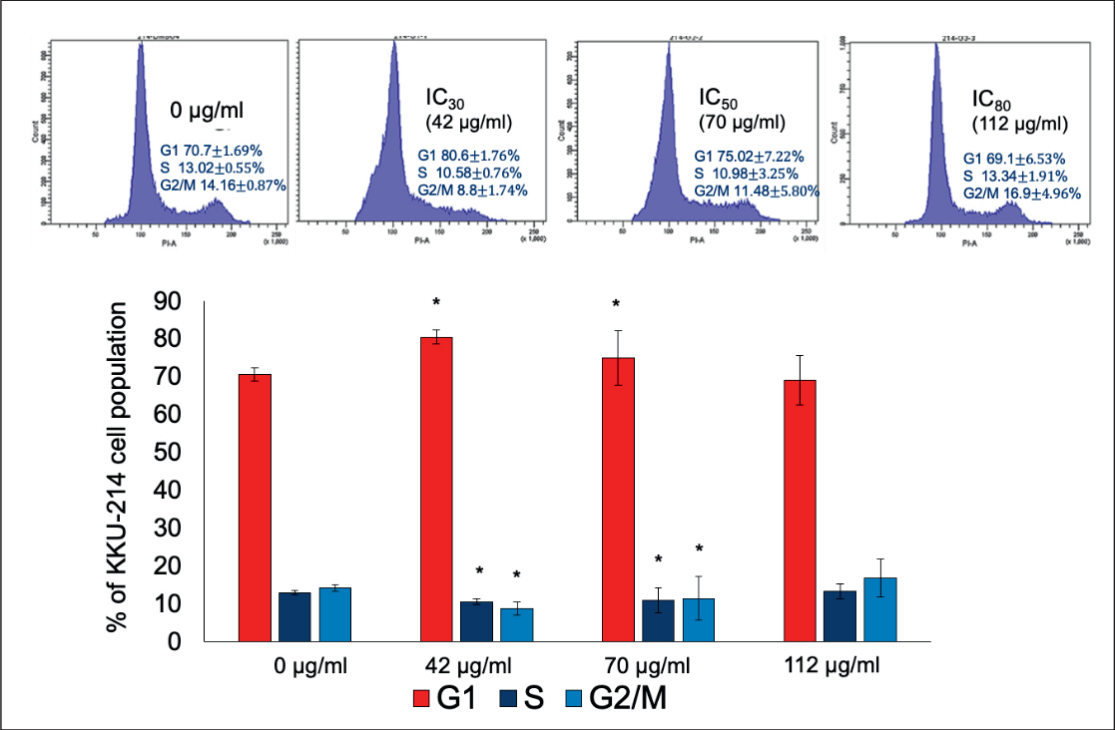
MAO-induced cell cycle arrest in KKU-214 as measured by flow cytometry. The cell cycle distribution pictures in each group are marked with a “*” to show a significant (*p < *0.05) difference from the control.

Using a previous technique, we observed that treating KKU-214 cells with MAO dramatically inhibited KKU-214 cell proliferation. The cells were examined by flow cytometry after their DNA had been labeled. The results indicated that at concentrations of 42 μg/ml G1 = 80.6% and 70 μg/ml G1 = 75.02%, MAO increased the percentage of cells at the G1 phase by 5–10% (in comparison to the untreated group 70.7%) and subsequently decreased them at S to G2/M phase (Fig. 6).

**Figure 7.**
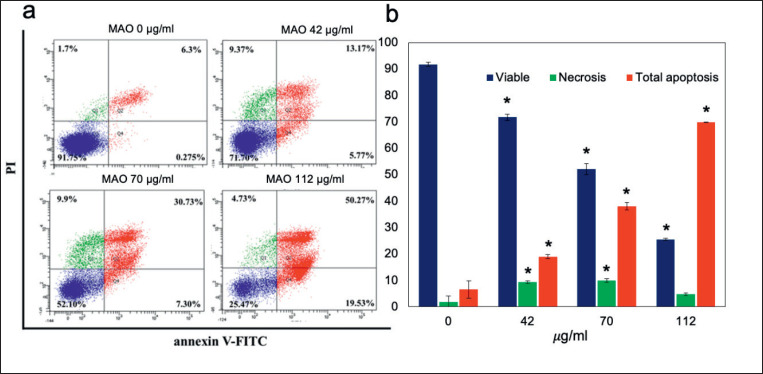
Flow cytometry-derived dot plot diagrams of apoptosis. All groups had viable cells in the left lower quadrant (FITC-/PI-), early apoptotic cells in the right lower quadrant (FITC+/PI-), and late apoptotic cells in the right upper quadrant (FITC+/PI+) *p < 0.05 compared to control.

**Figure 8.**
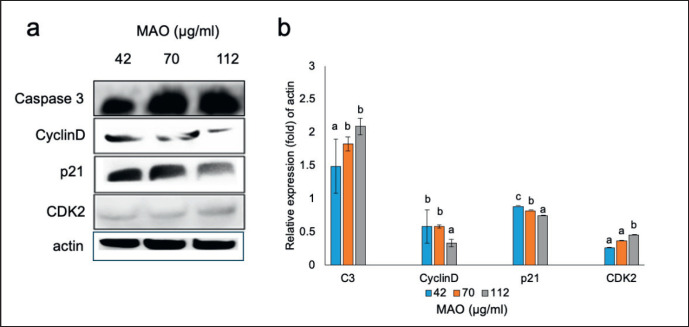
A representative Western blot analysis was conducted to determine the expression of Caspase-3, p21, Cyclin D, and CDK2 on KKU-214 with an internal control (actin) on treatment with MAO extract. The cumulative data for each assay was collected from three different trials and presented as mean SD (*n =* 3). ^a-c^*p* < 0.05 in the comparison.

### Effect of MAO extract on apoptosis in CCA cell line

MAO was tested to determine if it effectively inhibited cancer cell viability and induced cell apoptosis. Flow cytometry was used to assess apoptosis in cells using Annexin V-FITC labeling and PI. Figure 7 shows the total apoptotic cells of MAO were found to be 18.94% (IC_30_), 38.03% (IC_50_), and 69.80% (IC_80_). MAO extract induced apoptosis in KKU-M214 cells in a concentration-dependent manner.

### Effects of MAO on Caspase3, p21, Cyclin D, and CDK2 protein expression

The mechanism of apoptosis and cell cycle arrest related to protein Caspase-3, Cyclin D, p21, and CDK2 expression was investigated by Western blotting. MAO increased apoptosis by activating caspase-3 and down-regulating the phosphorylation of cyclin D, p21, and CDK2 expression in both time- and dose-dependent manners in KKU-214 cells (Fig. 8). These results are in consonance with the apoptosis result of flow cytometry analysis.

## Discussion

*Antidesma thwaitesianum* (MAO) is utilized in a variety of food and health items throughout the world. *In vitro* and *in vivo* anticancer activities of MAO have been described [6,7,17]. Polyphenols found in MAO extract, such as C3G, have antioxidant, inflammation-reducing, and anticancer properties. Cyanidin 3-glucoside in MAO extract, identified by HPLC, could suppress the development of cancer cells and trigger apoptosis. In addition, MAO extract showed significant phenolic and flavonoid contents, which resulted in anti-oxidant activity. This was similar to [7], who observed DPPH radical scavenging activity in berries. Inflammation resolution is also important because it promotes tissue repair following damage; inability to resolve causes prolonged inflammation, protracted tissue degradation, and increasing fibrosis [18]. Fibrosis is the accumulation of connective tissue to retain tissue form; enhanced fibrosis reflects a pathological condition [19].

Interestingly, MAO extracts possess C3G (anthocyanin), which acts as an anti-oxidant agent and possesses potent antioxidant effects as a result of its molecular structure and placement inside the plasma membrane. These factors support free radical neutralization, inhibiting inflammation in the bile duct and liver tissue of liver fluke infection. This corresponds with a previous study on the effects of anthocyanin complexes on periductal fibrosis [20]. The investigation into liver toxicity revealed no adverse effects of MAO at a dosage of 100 mg/kg for 1 month after therapy. The extract could also reduce liver damage caused by OV infection, as evidenced by decreased ALT and ALP levels, which correspond with histology. There is a capable defensive mechanism in the body that includes antioxidant enzymes with the ability to prevent oxidative damage, such as SOD, CAT, glutathione peroxidase, and nonenzymatic GSH. MDA is a free radical generated by the interaction of a free radical and a lipid, which can modify the structure of the cell membrane and subsequently induce mutations in DNA. SOD helps to maintain the balance between ROS production and removal by catalyzing the dismutation of superoxide radicals. MDA is a radical oxidative marker, whereas SOD is an endogenous antioxidant [21].

Catalase (CAT) is a key enzyme that prevents oxidative damage in cells by accelerating the breakdown of hydrogen peroxide into water and oxygen. Furthermore, glutathione and vitamins assist the cell’s protective enzyme system. In this study, MAO therapy significantly reduced MDA levels, enhanced GSH levels, restored SOD activity, and increased CAT activity, compared to the OV-infected group, similar to the study of 10 medicinal herbs [22]. The treatment of 100 mg MAO considerably restored the decreased CAT activity following OV infection. SOD, an endogenous scavenger that converts superoxide anion radicals into hydrogen peroxide, was greatly elevated in the MAO groups. The observed increase in SOD enzyme activity following MAO treatment might have been due to oxidative activation of enzyme proteins. As a result, an increase in SOD activity in treated hamster serum may imply a decrease in superoxide anion. GPx protects cells from oxidative stress, and increasing GSH as a cofactor resulted in less liver damage.

The cytotoxicity assay findings showed that MAO extracts suppressed the KKU-214 cell line similarly to Mulberry-induced cytotoxicity in breast cancer cells (MDA-MB-453) and inhibited HCT-8 and MCF-7 cells [23]. Additionally, cyanidin-3-glucoside inhibited the viability of MDA-MB-231 and BT-549 cells [24]. The cell cycle stage transition is controlled by the successive activation and deactivation of cyclin complexes and CDK-regulatory proteins [25]. MAO extracts reduced cell growth, caused cell cycle arrest at the G1 phase, and induced apoptosis in a dose-dependent manner. The results of this study correlated with the findings of tortilla and blue corn, which arrested the cell cycle of breast cancer (MDA-MB-453) and prostate cancer (LNCaP) cells in the G1 phase and triggered apoptosis [26]. Herein, we analyzed the regulatory proteins by western blotting and found that MAO extracts induced apoptosis by activation of caspase-3.

Additionally, through the internal mitochondrial pathway and the external death receptor pathway, anthocyanins can induce apoptosis in cancer cells [27]. MAO demonstrated G1-phase cell arrest due to reduced p21, Cyclin D, and CDK4, similar to a previous study on anthocyanins inhibiting tumor cell growth and apoptosis induction [25]. Cyclin D1 regulates the G_0_/G_1_ checkpoint of the cell cycle. A protein family known as CDKIs controls the activity of the CDK-cyclin complexes, leading to hypophosphorylation of cell cycle arrest and p21 binding to and inhibiting the activity of the cyclin A2‐CDK2 complex, thereby preventing cell progression [28].

The anti-inflammatory and anticancer properties of MAO extract indicated that it may be a good alternative drug for treating OV infections and preventing and inhibiting CCA progression in patients.

## Conclusion

In conclusion, our data highlight the potential advantages of MAO extract. The extracts contain Cyanidin 3-glucoside, which had anti-inflammatory, anti-oxidative stress, and hepatoprotective effects in hamster Opisthorchiasis *in vivo* and *in vitro* models. In addition, the extracts inhibited CCA cell (KKU-214) growth, induced G1 phase arrest, and apoptosis. These findings may open the way for developing cancer agents derived from natural products and investigating innovative ways for CCA therapy.

## List of Abbreviations

ABTS, 2,2’-azino-bis (3-ethylbenzthiazoline-6-sulphonic acid); ALP, alkaline phosphatase; ALT, alanine transaminase; BHT, butylated hydroxytoluene; BUN, blood urea nitrogen; CAT, catalase; C3, caspase 3; C3G, cyanidin-3-O-glucoside; CDK2, cyclin-dependent kinases 2; CCA, cholangiocarcinoma; DPPH, 2,2-diphenyl-1-picrylhydrazyl; EDTA, ethylenediamine tetraacetic acid; GSH, glutathione; HPLC, high-performance liquid chromatography; IC30, 30% maximal inhibitory concentration; IC50, 50% maximal inhibitory concentration; IC80, 80% maximal inhibitory concentration; MAO, *Antidesma thwaitesianum* fruits; OV, *Opisthorchis viverrini*; OVM, *Opisthorchis viverrini* plus MAO; MDA, Malondialdehyde; P21, cyclin-dependent kinase inhibitor p21; SOD, superoxide dismutase; SRB, sulforhodamine B; TFC, total flavonoid content; TPC, total phenolic content; TCA, trichloroacetic Acid.
